# Effect of the *Copaifera langsdorffii* Desf. Leaf Extract on the Ethylene Glycol-Induced Nephrolithiasis in Rats

**DOI:** 10.1155/2013/131372

**Published:** 2013-08-20

**Authors:** Rejane Barbosa de Oliveira, Eduardo Barbosa Coelho, Marina Rezende Rodrigues, Ana Rita de Mello Costa-Machado, João Paulo Barreto de Sousa, Andresa A. Berretta, Jairo Kenupp Bastos

**Affiliations:** ^1^Departamento de Ciências Farmacêuticas, Faculdade de Ciências Farmacêuticas de Ribeirão Preto, Universidade de São Paulo, Avenida do Café s/n, 14040-903 Ribeirão Preto, SP, Brazil; ^2^Departamento de Clínica Médica, Faculdade de Medicina de Ribeirão Preto, Universidade de São Paulo, Avenida Bandeirantes 3900, 14048-900 Ribeirão Preto, SP, Brazil; ^3^Apis Flora Industrial e Comercial Ltda., Rua Triunfo 945, 14020-470 Ribeirão Preto, SP, Brazil

## Abstract

The potential of the *Copaifera langsdorffii* leaves extract to prevent stone formation was analyzed by means of an ethylene glycol (EG) animal model of nephrolithiasis and an *in vitro* crystallization assay. Different doses of the *C. langsdorffii* leaves extract were administered to rats treated with EG. Urine biochemical parameters were quantified. CaOx deposits count and analysis of osteopontin expression were conducted on kidneys fixed in formalin. The *in vitro* assay was performed by turbidimetry. Phytochemical analyses of the extract were accomplished by HPLC-UV-DAD, and several compounds were isolated. *C. langsdorffii* leaf extract was able to avoid stone formation. The number of deposits was 50.30 ± 31.29 at the higher extract dose, compared to the value of 179.5 ± 45.96 achieved with the EG control. Significantly lower oxalate levels and OPN expression and increased citrate levels were observed after extract administration. In the *in vitro* assay, the extract diluted the formed crystals. Phytochemical analyses showed that the extract is rich in phenolic compounds that are capable of preventing stone formation. Thus, on the basis of our results, we suggest that the *C. langsdorffii* leaf extract has potential application in the prevention of kidney stone formation.

## 1. Introduction

Renal stones are a urological disorder that affects about six percent of the American population [1]. Nearly 80% of these stones consist of calcium oxalate (CaOx) and calcium phosphate [2]. CaOx supersaturation can culminate in infections, hemorrhages, and renal stones (nephrolithiasis), which in turn cause strong pain and discomfort in patients. Current treatments for nephrolithiasis include surgical intervention and the use of lasers for stones destruction [3]. However, recurrence after these treatments is common in most patients (about 10% at 1 year), and an effective drug that can eliminate these stones and avoid their recurrence does not exist [4].

Studies have demonstrated that some plant extracts are capable of ameliorating the clinical renal symptoms by dilution or elimination of the CaOx stones. Expressive results have been published for *Phyllanthus niruri *and green tea [5, 6]. Recently, we have reported on the potential of the *Copaifera langsdorffii* leaf extract for the treatment of renal stones in a model of CaOx calculi induced in the rat bladder [7].

*Copaifera langsdorffii* Desf. (Fabaceae) is a tree native to Brazil [8]. This species is known as copaiba and is used in folk medicine for the treatment of urinary disorders [9]. Pharmacological studies have attested to the efficacy of the copaiba oil resin and leaf extracts as anti-inflammatory, antinociceptive, antimicrobial, and wound healing agents [9–13]. Phytochemical studies carried out with the copaiba oil resin have revealed that it contains diterpenes, polyalthic acids, and sesquiterpenes [13–16]. Some of the compounds isolated from copaiba display different biological activities; for instance, (−)-copalic acid exhibits anticariogenic and antimicrobial effects [13, 14].

Although the composition of the copaiba oil resin and its biological actions have been well explored, the chemical composition of the leaf extracts of this plant and their potential biological activities remain unknown. Recently, our research group examined the chemical composition of the copaiba leaf hydroalcoholic extract. We detected several phenolic compounds, especially flavonoids, with a wide spectrum of biological actions [17]. Thus, based on our previous promising results concerning the chemical composition of the copaiba leaf extract [17] and its capacity to dilute CaOx stones implanted in the rat bladder [7], the aim of the present study was to analyze the potential of this extract for the treatment of renal stones in ethylene glycol-induced nephrolithiasis in rats. This model allows for the investigation of the efficiency of this extract in preventing kidney stone formation and/or facilitating stone elimination from this organ. An additional *in vitro* assay was carried out, in order to examine how the copaiba extract avoids CaOx crystallization and promotes crystals dissolution.

## 2. Material and Methods

### 2.1. Plant Material

*Copaifera langsdorffii* Desf. (Fabaceae) leaves were collected in November 2008 in the Campus of Ribeirão Preto of the University of São Paulo (USP) (21°10′S, 47°50′W, altitude 748 m), Brazil. A voucher specimen (SPFR 10120) was deposited in the SPFR herbarium from the University of São Paulo (USP), Brazil. The identification was confirmed by Prof. Dr. Milton Groppo Junior. 

### 2.2. Extract Preparation

The leaves were dried at 40°C, powdered (3.1 kg), and submitted to maceration in a 70% hydroalcoholic solution (3 × 72 h). The obtained extract was filtered, concentrated under vacuum, lyophilized, and kept in a freezer at –20°C until use (yield = 520 g) [7]. The product described herein is under patent protection no. PCT/BR2011/000090 (priority BR PI 1000802-0).

### 2.3. Phytochemical Extract Characterization

We have recently developed a validated HPLC-UV-DAD chromatographic method for the characterization and quantification of polar compounds in copaiba leaf hydroalcoholic extracts [17]. Briefly, 1.0 mL extract was filtered through Millex-LCR-PTFE (Millipore, Bedford, MA, USA, 0.45 *μ*m × 13 mm i.d.), and 10 *μ*L of the filtered extract was injected into the HPLC system (Shimadzu SCL-10Avp, Kyoto, Japan, coupled to a Shimadzu SPD-M10Avp photodiode array detector-DAD). The analytical chromatography was carried out using two monolithic columns linked in series (Onyx 100 × 4.6 mm-C_18_ Phenomenex), coupled to a precolumn. The mobile phase consisted of water (A) and acetonitrile (B). The elution program was 5-6% of B for 1 min, 6–8% of B (min. 1-2), 8–10% of B (min. 2–5), 10–15% of B (min. 5–12), 15% of B (min. 12–22), 15–25% of B (min. 22–27), 25% of B (min. 27–35), 25–40% of B (min. 35–39), 40% of B (min. 39–42), 40–100% of B (min. 42–47), and 100% of B for 1 min, and 12 additional minutes were allowed for the system to return to the initial conditions. The flow rate was 1.0 mL/min. The spectral data were collected from 256 to 366 nm, and the chromatogram was plotted at 257 nm. All the peaks were assigned according to the retention times, coelution with authentic standards, and UV spectra of the compounds under the same chromatographic conditions.

### 2.4. Isolation of Compounds

Procedures to purification of several compounds from copaiba leaf hydroalcoholic extracts were previously published by our research group [18]. Briefly, the crude hydroalcoholic extract from leaves (50 g) was dissolved in 1 L of methanol 90% and partitioned with *n-*hexane, chloroform, and ethyl acetate. The ethyl acetate fraction (10 g) was chromatographed on classical silica gel column using a crescent gradient of *n-*hexane from ethyl acetate. The resultant fractions were monitored by TLC, and some were chosen to further purification. Both I (3 g) and III (1 g) fractions were rechromatographed on classical column. The fraction V (0.3 g) was treated with ethereal diazomethane, and the methylated sample was submitted to classical column using an isocratic system of *n*-hexane-ether 95 : 5%. The fraction VII (1 g) was purified by semipreparative HPLC using a Shimadzu liquid chromatography (Kyoto, Japan), equipped with an LC-AD solvent pump unit, an SCL-10A system controller, and an SP-M10A diode array detector Shimadzu, operating with chromatopak C-R6A integrator (Kyoto, Japan) and reverse-phase Shim-Pack CLC-ODS (C18) column (Shimadzu, Tokyo, Japan; 250 × 20 mm i.d.). The mobile phase consisted of water (A) and methanol (B). The elution program was 30% B, followed by a linear gradient to 100% of B after 30 min, at a flow of 6.0 mL/min. High-resolution ESIFORMS data of main compounds were obtained on an Agilent 6210 mass spectrometer. NMR spectra were recorded on a Varian Mercury 400 BB spectrometer (400 MHz for ^1^H and 100 MHz for ^13^C). Samples were dissolved in CDCl_3_ or DMSO-*d* depending on polarity of the isolate compounds. TMS was used as internal reference.

### 2.5. Animals

Adult male Wistar rats (200–250 g) from the animal facility of the Faculty of Pharmaceutical Sciences of Ribeirão Preto of the University of São Paulo (FCFRP-USP) were maintained under standard laboratory conditions (25 ± 2°C at 40–60% relative humidity in a 12 h light-dark cycle), with free access to water and a balanced ration Nuvilab CR-1 from Nuvital Nutrientes S/A (Brazil). The study was approved by the Institutional Animal Ethics Committee of FCFRP-USP (protocol number: 10.1.1800.53.3, it was approved on February 02, 2011).

### 2.6. Experimental Protocol

Animals were divided into nine groups (*n* = 9 animals/group) and housed individually in propylene cages. Group C comprised the untreated control, which received filtered water; group EG was administered ethylene glycol (EG) 1% (V/V), and group CP160 received copaiba leaf extract at 160 mg. Both EG and the extract were diluted in 100 mL water and administered to the animals in their drinking water for 28 days. Additional groups were divided into two categories: denominated prophylactic and post-stone treatment groups. In the case of the prophylactic group, animals concomitantly received copaiba leaf extract at different concentrations (group CP40+EG: 40 mg, group CP80+EG: 80 mg, and group CP160+EG: 160 mg) and EG 1% diluted in 100 mL of their drinking water for 28 days, on a daily basis. The poststone treatment group received only EG 1% for 14 days; after this period, the animals belonging to this group received EG 1% plus copaiba leaf extracts at different concentrations (group EF+CP40: 40 mg, group EF+CP80: 80 mg, group EF+CP160: 160 mg), which were diluted in 100 mL of their drinking water for additional 14 days, on a daily basis (total of 28 days). The fluid intake was measured daily, and a new solution of EG or EG and extracts were also prepared on a daily basis. At the end of the experiment 24-hour urine samples were collected for biochemical analyses. The body weight of the animals was recorded on both the first and the last days of the experiment. Subsequently, animals were euthanatized in a CO_2_ chamber, and their kidneys were removed and fixed in buffered formalin.

### 2.7. Urinary Variables Measurement

Phosphorus, magnesium, calcium, and uric acid were analyzed by using commercial kits (Labtest Diagnostics, Brazil). Urinary oxalate was determined by direct precipitation followed by titration, as previously described [19]. Urinary citrate was measured by means of a previously described colorimetric assay [20]. Urinary pH was obtained with the aid of pH stripes (Chemco, Brazil).

### 2.8. Oxalate Crystal Deposits Count

Kidneys fixed in formalin were submitted to a standardized protocol of histological slide preparation. Seven micrometer sections of deparaffinized kidneys were stained in silver nitrate for 1 h, under exposure to incandescent light, followed by counterstaining with a hematoxylin-eosin preparation. CaOx crystals formation and retention were quantified by counting the number of CaOx deposits stained with silver nitrate per longitudinal kidney section.

### 2.9. Immunohistochemical Staining

Deparaffinized 7 *μ*m sections of the formalin-fixed kidneys were subjected to antigen unmasking by heat treatment in sodium citrate buffer 10 mM (pH 6.0) heated at 95°C, for 10 min. Endogenous peroxidases were blocked with hydrogen peroxide 0.5% (V/V) in PBS (pH 7.2), for 10 min. Further slide preparations were performed by using an immunohistochemistry staining system kit purchased from Santa Cruz Biotechnology Europe (sc-2017). To this end, slides were incubated with a monoclonal antibody against osteopontin (OPN), which was followed by a biotinylated secondary antibody, according to the manufacturer's instructions. The slides were then counterstained with methyl green. Controls were accomplished by omitting the primary antibody. The OPN expression was calculated by counting the number of positive renal tubules per longitudinal kidney section.

### 2.10. *In Vitro* Crystallization Assay

The *in vitro* effect of the copaiba leaf extract on the nucleation and aggregation of calcium oxalate monohydrate crystals was studied by using a previously described turbidimetric method [21]. Briefly, calcium oxalate monohydrate crystallization was obtained by mixing 12.5 mL calcium chloride (8 mmol/L) into 12.5 mL sodium oxalate (1 mmol/L) and sodium acetate/acetic acid buffer 10 mmol/L (pH 5.7). Two distinct tests were accomplished: (1) crystals formation inhibition test and (2) preformed crystals dissolution test. In the first test, solutions containing the copaiba leaf extract at concentrations of 0.3, 0.7, or 1.0 mg/mL were added concomitantly to the solution containing the crystallization reagents at time 0 h (before crystallization). A control test was carried out using the same amount of vehicle employed for dilution of the leaf extract (ethanol 70%). The samples were kept under 500 rpm agitation at 37°C, for 24 h. As for the dissolution test, crystals formation was induced under the same conditions mentioned previously, for 24 h. After crystals formation, solutions of the copaiba leaf extract at 0.3, 0.7, or 1.0 mg/mL were added; these samples were used as control (time 0 h). The absorbance at 620 nm and the counter of oxalate crystals on light microscope were measured before (time 0 h, control) and 24 h after leaf extract addition. The tests were performed as independent triplicates for each concentration.

### 2.11. Statistical Analyses

The differences among experimental and control groups were determined using the statistical software GraphPad Prism 5 (GraphPad Software, Inc., USA). Data are represented as the mean ± S.E.M. and were analyzed by one-way ANOVA followed by Dunnett's test, in order to compare all the treated groups with control groups in the case of the *in vivo* assays. Two-way ANOVA followed by Bonferroni's test was used in the case of the *in vitro* assay. In all analyses *P* value less than 0.05 was considered statistically significant.

## 3. Results

**Figure 1:**
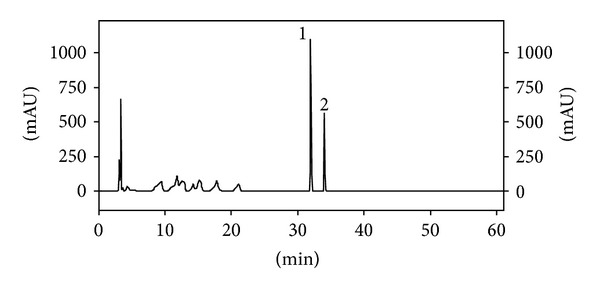
Chromatographic profile recorded at 257 nm of the hydroalcoholic extract from *C. langsdorffii *leaves. 1: quercitrin and 2: afzelin.

The chromatographic procedures of the *C. langsdorffii *leaves extract allow the isolation of seven compounds [18]. The structural elucidation of the isolated compounds was made by their high-resolution mass spectra and ^1^H and ^13^C NMR data compared to the literature. The fraction I furnished the kaurenoic acid, caryophyllene oxide, and kaurenol. The compound 2-hydroxy-*ent*-labda-7,13-dien-15-oic acid was isolated from fraction III; the flavonoids quercitrin and afzelin were purified from fraction VII, while the methylated sample furnished the compound ethyl 4-metoxycinnamate. Analyses of the *C. langsdorffii* leaf hydroalcoholic extract by reverse phase HPLC-UV-DAD allowed for identification of quercitrin and afzelin as the major flavonol compounds (Figure 1), and for this reason these compounds were chosen to method validation procedures [17]. 

The general variables analyzed in the *in vivo *study are shown in Tables 1 and 2. Fluid intake and urine output increased in groups EG and in all the post-stone treatment groups, compared to the untreated control group C. More specifically, the following fluid intake and urine output results were, respectively, achieved (Table 2): EG (70.84 ± 5.29; 22.76 ± 1.65 mL/24 h), EF+CP40 (68.36 ± 8.07; 20.35 ± 2.49 mL/24 h), EF+CP80 (69.84 ± 4.42; 24.69 ± 2.95 mL/24 h), and EF+CP160 (70.37 ± 5.99; 21.27 ± 3.90 mL/24 h) and group C (37.98 ± 3.41 mL/24 h; 9.73 ± 1.29 mL/24 h). Urinary pH decreased in EG, in the prophylactic group CP40+EG, and in the post-stone prophylactic groups EF+CP40, EF+CP80, and EF+CP160 (6.00 ± 0.00) in relation to group C (7.00 ± 0.00), Tables 1 and 2. There were no statistical differences among the groups in terms of animal body weight. 

There were no statistically significant alterations in the calcium, magnesium, and phosphate levels in any of the groups, compared to group C (Tables 3 and 4). However, oxalate levels rose in group EG (2.31 ± 0.21 mg/24 h) and in all the post-stone treatment groups, more specifically EF+CP40 (2.08 ± 0.24 mg/24 h), EF+CP80 (1.75 ± 0.17 mg/24 h), and EF+CP160 (1.72 ± 0.23 mg/24 h), compared to group C (0.63 ± 0.16 mg/24 h). 

Reduced citrate levels were verified in groups with augmented oxalate levels (Table 4), compared to the untreated control groups (C: 33.82 ± 5.89 mg/24 h; EG 18.73 ± 2.72 mg/24 h; EF+CP40: 23.85 ± 2.76 mg/24 h; EF+CP80: 23.66 ± 3.39 mg/24 h; and EF+CP160, 20.87 ± 2.68 mg/24 h). 

Significantly higher uric acid levels were observed in EG (Table 2), whereas normal uric acid levels were detected in all the animals treated with the copaiba leaf extract.

**Figure 2:**
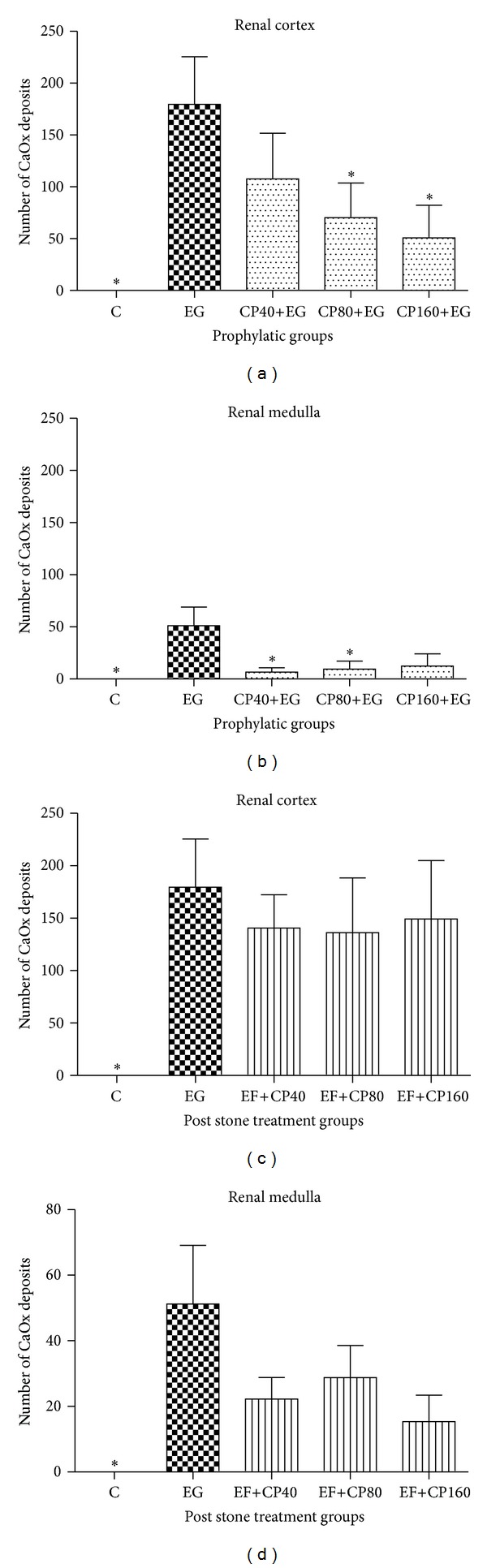
Quantification of renal CaOx deposits on silver nitrate-stained kidney sections ((a) and (b)). Renal cortex and medulla of the prophylactic groups ((c) and (d)). Renal cortex and medulla of the poststone treatment groups. *P < 0.05 compared to group EG.

**Figure 3:**
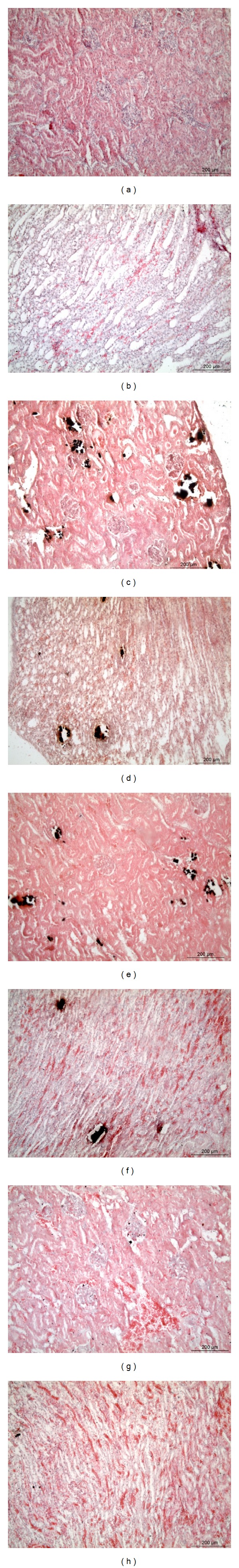
Light microscopy of the rat renal cortex and medulla revealed CaOx deposits in the lumen of renal tubules stained with silver nitrate. Copaiba leaf extract group: no calcium oxalate or histological alterations were observed ((a) and (b)). EG 1% group: glomerular degeneration, tubular necrosis, amorphous deposits in the tubular lumen, and several calcium oxalate deposits were detected ((c) and (d)). Post-stone treatment group: glomerular and tubular degenerations and calcium oxalate deposits were similar to those verified for EG 1% group ((e) and (f)). Prophylactic treatment group: glomerular and tubular degenerations were decreased in relation to EG 1% group, as well as the number of calcium oxalate deposits ((g) and (h)). (100x magnification).

Calcium deposits were not observed in the kidneys of untreated control animals (group C) or in the group that received only the copaiba leaf extract (group CP160) (Figures 2 and 3). Reduced numbers of CaOx deposits were detected in all the prophylactic treatment groups. However, in the cortex region, this decrease was statistically significant only in groups CP80+EG (70.40 ± 33.22) and CP160+EG (50.30 ± 31.29), compared to the EG control group (EG: 179.5 ± 45.96). In the case of the medullar region, there was a significant decrease in the number of deposits only in groups CP40+EG (6.67 ± 4.12) and CP80+EG (9.47 ± 7.74), compared to group EG (51.20 ± 17.91). The post-stone treatment groups did not perceive a significant reduction in the number of CaOx deposits (Figures 2 and 3).

The renal tissue of the animals belonging to EG was severely damaged. Indeed, glomerular degeneration and enlarged renal tubules with amorphous deposit in their lumen (Figures 3(c) and 3(d)) were observed. In the post-stone treatment and prophylactic groups, it was possible to note an improvement of these tissues, especially in the prophylactic groups (Figures 3(g) and 3(h)).

**Figure 4:**
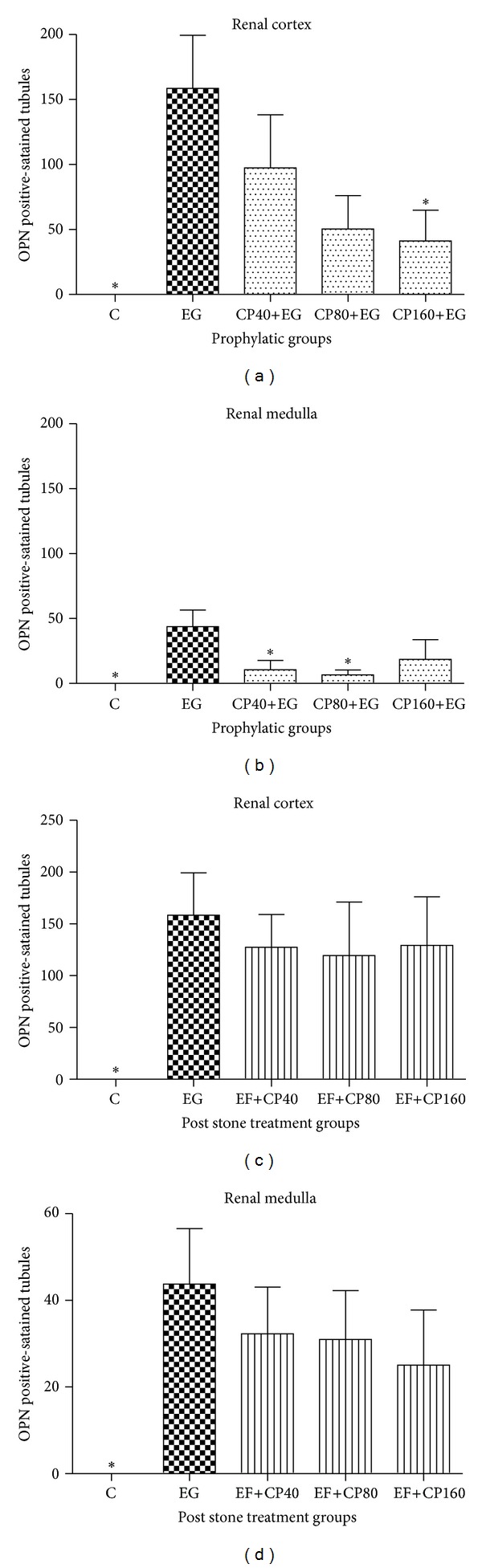
Quantification of the OPN positive-stained renal tubules in kidney sections ((a) and (b)). Renal cortex and medulla of the prophylactic groups ((c) and (d)). Renal cortex and medulla of the post-stone treatment groups. *P < 0.05 compared to group EG.

**Figure 5:**
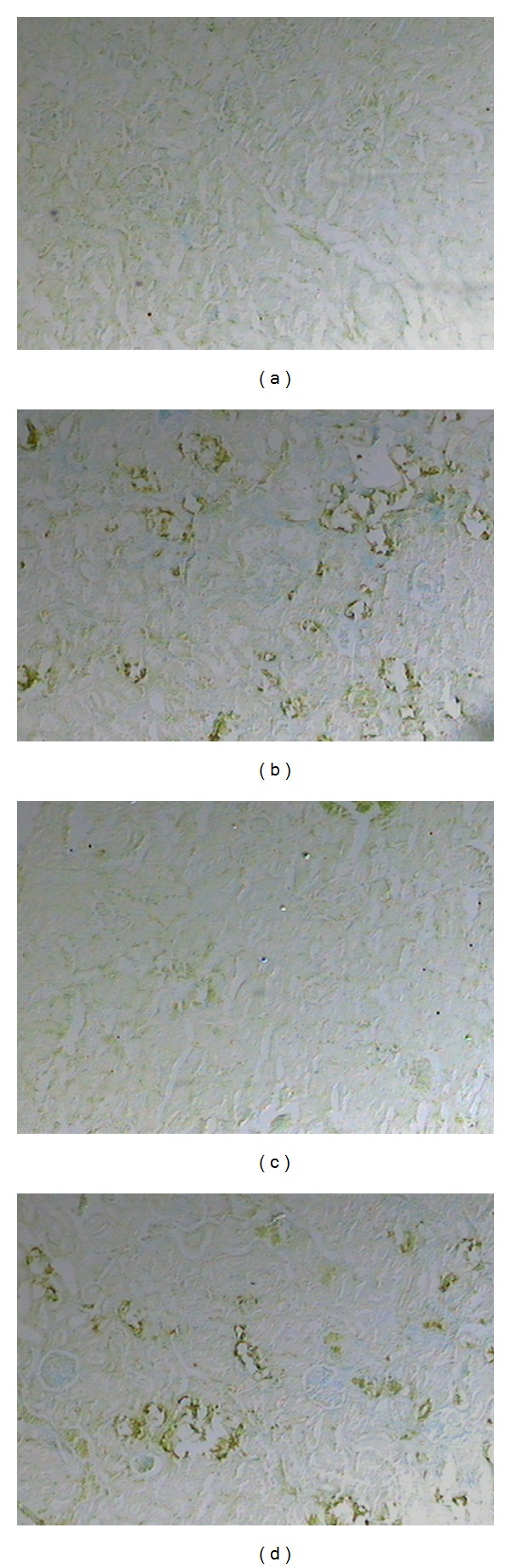
Immunohistochemical analyses of renal tubules stained for OPN. Copaiba leaf extract group: no OPN synthesis was detected (a). EG 1% group: increased OPN synthesis in the renal tubules epithelium was observed (b). Prophylactic group: OPN expression decreased in relation to the EG 1% group (c). Post-stone treatment group: increased OPN synthesis in renal tubules is similar to that in EG 1% group (d). (40x of magnification).

As in the case of the quantification of CaOx deposits, only the groups that received the prophylactic treatment presented significantly lower OPN expression (Figures 4 and 5). Significantly diminished OPN expression was seen in the cortex region of the animals belonging to group CP160+EG (41.20 ± 23.75), compared to group EG (158.5 ± 40.83). 

Concerning the *in vitro* assay (Table 5), the effect of the copaiba leaf extract was statistically significant for all the tested doses in terms of crystals formation prevention (100% inhibition). In the case of the crystals dissolution test, the results were significant at doses of 0.7 (79.7% dissolution) and 1.0 mg/mL (100% dissolution).

## 4. Discussion

The EG model has been successfully used by various authors in the study of nephrolithiasis in rats [5, 22, 23]. EG is metabolized to oxalic acid by different hepatic enzymes, with subsequent precipitation and growth of insoluble CaOx crystals in the renal tubule epithelium [24]. Biochemical analysis of the urine of animals treated with EG reveals several disorders, including enhanced water intake and urine output, hypercalciuria, hyperoxaluria, hypocitraturia, hypomagnesiuria, and hyperuricosuria [23]. These conditions result in abnormal urinary pH and calcium, oxalate, citrate, magnesium, potassium, and uric acid excretion [23].

In the present study, EG administration modified several of the above-mentioned parameters in rats. Moreover, the concomitant administration of the copaiba leafextract led to the normalization of some variables such as fluid intake, urine output, and pH. Increased uric acid excretion is usually observed in nephrolithiasic patients and animals treated with EG. Here, normal uric acid levels were verified in all the animals treated with the copaiba leaf extract, which can contribute to the reduction of the risk of stone formation. Additionally, in a previous study published by our research group, we have demonstrated that the levels of sodium and potassium did not change after the administration of the hydroalcoholic of the *C. langsdorffii *to the animals [7].

Most importantly, oxalate levels were normal in the prophylactic groups. Hyperoxaluria is a more significant risk factor in the pathogenesis of renal stones than hypercalciuria or increased levels of other minerals [25]. The normalization of the oxalate urinary excretion levels in the prophylactic groups indicated that this can be a relevant protective effect of the copaiba leaf extract in terms of stone production. Additionally, the prophylactic administration of the copaiba leaf extract brought the urinary citrate excretion levels near to normal, suggesting that animals had an improvement in citrate regulation. Citrate is an inhibitor of crystallization, because it can reduce the calcium oxalate saturation by forming a complex with calcium [25]. In fact, hypocitraturia is a major metabolic abnormality in patients with renal stones [26]. These effects on the oxalate and citrate levels may have contributed to the smaller number of CaOx deposits and the lower OPN expression detected in all the prophylactic groups. OPN has been identified as one of the most important macromolecules associated with CaOx crystals retention in kidneys [22]. As in the case of CaOx deposit quantification, groups that received the prophylactic treatment exhibited significantly decreased OPN expression. OPN is localized at specific sites in the kidney and is generally restricted to renal epithelial cells and tubules in the cortex. However, in nephrolithiasis there is elevated OPN synthesis in the renal cortex and medulla, where it is closely associated with crystal deposits [23].

Regarding the safety of using copaiba leaf extract in animal protocols, it was reported that the extract does not change levels of sodium, potassium, and creatinine in both serum and urine samples [7], which indicate that the extract did not affect the renal function [7]. Moreover, it was demonstrated that the hydroalcoholic extract of *C. langsdorffii* has no genotoxic effect in the mouse erythrocyte micronucleus test [27].

The *in vitro* assay was performed with the objective of gaining more information about the potential of the copaiba leaf extract to both prevent crystals formation and dilute the formed crystals. In this experiment, approximately 30 min after mixing the calcium chloride and sodium oxalate solutions there was maximum increase in the optical density, which reflects the maximum crystal nucleation rate. After the solution reached equilibrium, the optical density became progressively lower, due to the diminished turbidity that resulted from the utilization of disperse particles in crystal aggregation [21]. In other words, decreased optical density was the outcome of enhanced crystal aggregation. In our experiment, copaiba leaf extract addition culminated in an inverse effect. The optical density increased with time, compared to the control. This is indication that the extract prevented crystal aggregation and dissolved the formed crystals *in vitro*.

Phytochemical studies regarding the chemical constituents of copaiba leaves are scarce. However, we have purified several compounds from the polar extract of copaiba leaves, including kaurenoic acid, quercetin, and afzelin [18]. Kaurenoic acid is a diterpene with potent anti-inflammatory activity by the inhibition of the inducible nitric oxide synthase (iNOS) and ciclooxigenase-2 (COX-2) expression [28, 29]. COX-2 inhibitors such celecoxib have protective effects against transient renal dysfunction that occurs due to the shock waves in the shock waves lithotripsy treatment [30]. Additionally, it has been demonstrated that kaurenoic acid produces a vasorelaxation by inhibition of the Ca^2+^ influx [31]. Calcium channel blockers associated with shock waves lithotripsy increased the stone expulsion rates [32]. HPLC method for quantification of the flavonols present in copaiba leaves allowed the identification of quercitrin and afzelin as major compounds in the hydroalcoholic copaiba leaves extract [17]. Flavonol quercitrin also exists in *Phyllanthus niruri*, which is used in the treatment of kidney stones in traditional Indian Ayurvedd, and Chinese medicine [33]. Flavonoids and other phenolic compounds are potent antioxidant agents. They prevent renal stones formation since it has been demonstrated that oxidative damage is an essential factor regarding CaOx crystals retention in the tubular epithelium [22]. Additionally, it has been reported that quercetin inhibits the oxidative damage induced by oxalate in renal tubular cells and renal tissues and that it prevents renal stones formation in an animal model [34]. This information suggests that the compounds that we have identified in the copaiba leaves present action in the renal protective effect observed.

## 5. Conclusions

The results of this study indicate that the copaiba leaf extract has a prophylactic potential in terms of preventing kidney stones formation. Thus, on the basis of our results and literature information about the compounds present in the copaiba extracts, it can be inferred that the copaiba leaf extract has potential application in the prevention of stones recurrence in humans. However, the exact mechanism of this protective effect is not completely understood. Further studies should be conducted to better characterize the mechanism of action of *C. langsdorffii* in preventing kidney stones formation as well as to evaluate possible toxic effects in a regime of long-term oral administration.

## Figures and Tables

**Table 1:** General variables data for the prophylactic groups.

Group	Body weight (g)	Fluid intake (mL/24 h)	Urine output (mL/24 h)	Urinary pH
C	419.3 ± 84.08	37.98 ± 3.41	9.73 ± 1.29	7.00 ± 0.00
EG	302.5 ± 67.86	70.84 ± 5.29*	22.76 ± 1.65*	6.00 ± 0.00*
CP160	424.0 ± 104.8	35.64 ± 3.41	10.41 ± 1.86	7.30 ± 0.18
CP40+EG	363.0 ± 58.78	50.76 ± 4.41	18.81 ± 1.88	6.00 ± 0.00*
CP80+EG	409.8 ± 84.94	49.87 ± 3.05	15.51 ± 2.72	6.43 ± 0.29
CP160+EG	426.5 ± 53.95	45.27 ± 2.68	15.24 ± 2.72	7.00 ± 0.41

*P < 0.05 compared to group C.

**Table 2:** General variables data for the poststone treatment groups.

Group	Body weight (g)	Fluid intake (mL/24 h)	Urine output (mL/24 h)	Urinary pH
C	419.3 ± 84.08	37.98 ± 3.41	9.73 ± 1.29	7.00 ± 0.00
EG	302.5 ± 67.86	70.84 ± 5.29*	22.76 ± 1.65*	6.00 ± 0.00*
CP160	424.0 ± 104.8	35.64 ± 3.41	10.41 ± 1.86	7.30 ± 0.18
EF+CP40	396.0 ± 53.95	68.36 ± 8.07*	20.35 ± 2.49*	6.00 ± 0.00*
EF+CP80	416.0 ± 44.33	69.84 ± 4.42*	24.69 ± 2.95*	6.00 ± 0.00*
EF+CP160	365.0 ± 38.94	70.37 ± 5.99*	21.27 ± 3.90*	6.00 ± 0.00*

*P < 0.05 compared to group C.

**Table 3:** Urine biochemical parameters for the prophylactic groups.

	Oxalate (mg/24 h)	Citrate (mg/24 h)	Calcium (mg/24 h)	Magnesium (mg/24 h)	Phosphate (mg/24 h)	Uric acid (mg/24 h)
C	0.63 ± 0.16	33.82 ± 5.89	1.77 ± 0.50	2.33 ± 0.33	7.12 ± 0.84	1.17 ± 0.13
EG	2.31 ± 0.21*	18.73 ± 2.72*	2.54 ± 0.68	1.88 ± 0.39	9.31 ± 1.08	2.23 ± 0.29*
CP160	0.75 ± 0.24	32.95 ± 4.42	1.92 ± 0.50	2.61 ± 0.32	7.23 ± 1.20	1.26 ± 0.36
CP40+EG	0.59 ± 0.18	25.99 ± 3.64	2.50 ± 0.65	1.98 ± 0.06	9.10 ± 0.88	1.81 ± 0.22
CP80+EG	1.27 ± 0.29	31.07 ± 2.66	2.06 ± 0.42	2.37 ± 0.27	8.24 ± 1.03	1.50 ± 0.20
CP160+EG	0.99 ± 0.17	32.37 ± 3.60	2.22 ± 4.42	2.19 ± 0.17	7.66 ± 1.03	1.53 ± 0.23

*P < 0.05 compared to group C.

**Table 4:** Urine biochemical parameters for the poststone treatment groups.

	Oxalate (mg/24 h)	Citrate (mg/24 h)	Calcium (mg/24 h)	Magnesium (mg/24 h)	Phosphate (mg/24 h)	Uric acid (mg/24 h)
C	0.63 ± 0.16	33.82 ± 5.89	1.77 ± 0.50	2.33 ± 0.33	7.12 ± 0.84	1.17 ± 0.13
EG	2.31 ± 0.21*	18.73 ± 2.72*	2.54 ± 0.68	1.88 ± 0.39	9.31 ± 1.08	2.23 ± 0.29*
CP160	0.75 ± 0.24	32.95 ± 4.42	1.92 ± 0.50	2.61 ± 0.32	7.23 ± 1.20	1.26 ± 0.36
EF+CP40	2.08 ± 0.24*	23.85 ± 2.76*	2.21 ± 0.36	1.71 ± 0.16	9.09 ± 1.40	2.06 ± 0.16
EF+CP80	1.75 ± 0.17*	23.66 ± 3.39*	2.03 ± 0.31	1.83 ± 0.39	9.15 ± 1.33	2.12 ± 0.29
EF+CP160	1.72 ± 0.23*	20.87 ± 2.68*	2.54 ± 0.36	1.73 ± 0.29	10.09 ± 1.30	1.96 ± 0.26

*P < 0.05 compared to group C.

**Table 5:** *In vitro* crystallization assay.

	% inhibition of crystals formation	% dissolution of formed crystals
Control	0.0 ± 0.00	0.0 ± 0.00
0.3 mg/mL	100.0 ± 0.01*	15.3 ± 0.02
0.7 mg/mL	100.0 ± 0.01*	79.7 ± 0.01*
1.0 mg/mL	100.0 ± 0.02*	100.0 ± 0.01*

*P < 0.05 compared to the control group. (Two-way ANOVA followed by Bonferroni's multiple comparison test). Results were obtained at 24 h after extract addition.
